# Production and characterization of pellets with sewage sludge for use as an energy and environmental alternative

**DOI:** 10.1007/s11356-026-37914-6

**Published:** 2026-06-10

**Authors:** Bárbara Zanini, Samuel Nelson Melegari de Souza, Ricardo Sonsim de Oliveira, Edson Antonio da Silva, Lais da Silva Pego Hericks, Paulo Rodrigo Stival Bittencourt

**Affiliations:** 1 Center for Exact Sciences and Technologies, State University of West Paraná, Cascavel, Paraná, Brazil; 2 Federal Institute of Paraná, Cascavel, Paraná, Brazil; 3 Center for Engineering and Exact Sciences, State University of West Paraná, Toledo, Paraná, Brazil; 4 Federal Technological University of Paraná, Medianeira, Paraná, Brazil

**Keywords:** Sludge, Wastes, Pellets, Alternative fuel, Biomass, Energy

## Abstract

This study investigates the production and characterization of pellets incorporating sewage sludge as a partial substitute for lignocellulosic biomass, aiming at its valorization as an alternative energy source and environmentally sustainable solution. Pellets were produced using different proportions of sewage sludge and Pinus wood powder (0%, 25%, 50%, 75%, and 100%) in a pilot-scale pellet mill (1,500 kg·h⁻^1^). The samples were characterized in terms of moisture content, ash content, volatile matter, fixed carbon, higher calorific value (HHV), elemental composition (TXRF), thermal behavior (TGA), and gaseous emissions during pyrolysis (EGA-FTIR). Moisture content ranged from 1.89 to 6.76%, complying with ISO 18134–2 standards. Ash content increased proportionally with sludge incorporation (0.85 to 29.96%), reflecting the inorganic fraction of the residue. The HHV varied between 14.45 and 19.01 MJ·kg⁻^1^, with formulations containing up to 50% sludge meeting the ISO 18125 requirement (≥ 16.56 MJ·kg⁻^1^). Thermogravimetric analysis indicated increased thermal stability with higher sludge content, attributed to elevated mineral matter. Gas analysis revealed the predominance of CO₂, CH₄, and oxygenated compounds, with no detection of toxic gases at critical levels under the evaluated conditions. The results demonstrate that the incorporation of sewage sludge up to 50% in pellet formulations is technically feasible for energy applications, contributing to waste management strategies and circular economy practices while maintaining acceptable fuel properties.

## Introduction

Periodically, various types of wastes are generated and disposed of in landfills. Sanitary sewage can be defined, according to NBR 9648, as the liquid dumping formed from domestic and industrial sewage, infiltration water (coming from the subsoil, undesirable to the separator system and infiltrating the pipes) and the parasitic pluvial contribution, absorbed by the sewage collection network (ABNT 1986). It is one of the wastes currently disposed of in the landfill, thus being one of the environmental and financial problems of sanitation companies.

At the same time, sludge from wastewater treatment plants, considered a form of biomass, has gained increasing attention as a potential feedstock for energy recovery. Recent studies have focused on advanced thermochemical conversion processes, such as pyrolysis, hydrothermal carbonization, and integrated waste-to-energy systems, aiming to produce biofuels and other value-added products (Ruiz-Gómez et al. 2017; Toklu 2017).

Biomass, particularly in solid form, remains one of the most widely used renewable energy sources worldwide. Nevertheless, despite these advances, most sewage sludge valorization technologies are still restricted to laboratory or pilot-scale applications, highlighting the need for scalable and economically viable solutions for large-scale implementation.

Another study conducted by Oliveira et al. (2017) investigated the production and characterization of sewage sludge briquettes for use as fuel. The authors analyzed the optimal conditions and proportions required for briquette production, considering operational aspects, transport, storage, and energy performance. The results indicated that sewage sludge presents a calorific value comparable to other materials commonly used in briquette manufacturing, highlighting its potential as a viable raw material for energy generation. However, due to the limited commercial demand for briquettes, the present study focuses on pellet production in order to enhance the marketability and wider commercialization of this material.

In this context, pelletization emerges as a promising alternative. Pellets are small, dense cylindrical units typically produced from lignocellulosic biomass through compaction processes, and are widely used for thermal and electrical energy generation (Dias et al. 2012; Garcia et al. 2013). Compared to raw biomass, pellets offer several advantages, including improved storage and handling, higher bulk density, enhanced calorific value, ease of transport, material standardization, and reduced environmental impact (Loução 2008).

Furthermore, pellet quality must meet strict physical, mechanical, and energetic standards to ensure consistent performance in energy systems. Standardization is essential not only for national quality certification but also for access to international markets, which increasingly demand high-quality densified biomass products (Spanhol 2015).

Thus, considering the need for sustainable waste management and energy recovery, this study aims to produce and characterize pellets using sewage sludge and Pinus wood residues in different proportions (0%, 25%, 50%, 75%, and 100%).

The pellets were produced using a Rotex Master pellet mill (1,500 kg·h⁻^1^) and characterized in terms of moisture content, ash content, volatile matter, fixed carbon, calorific value, elemental composition (TXRF), thermal stability (TGA), and pyrolysis gas emissions (EGA-FTIR).

In addition, this approach contributes to waste valorization by promoting the reuse of wood residues and sewage sludge for energy generation. This aligns with the United Nations Sustainable Development Goals, particularly Goal 12 (Responsible Consumption and Production), which aims to substantially reduce waste generation through prevention, reduction, recycling, and reuse by 2030 (UN 2015). In Brazil, this issue is especially relevant, given that more than 990 thousand tons of dry sewage sludge are produced annually and are predominantly disposed of in landfills at an average cost of R$ 274.00 per ton (Sanepar 2023).

## Materials and methods

### Collection and preparation of raw materials

Sewage sludge samples were collected from a wastewater treatment plant (WWTP) located in Cascavel, Paraná, Brazil. The sampling procedure was carried out at different points of the decantation basin to ensure representativeness of the material. Approximately 90 kg of sludge were collected and subsequently subjected to drying and grinding processes in order to obtain a homogeneous material with controlled moisture content (10–12%) and suitable particle size for pelletization. Pinus wood powder was supplied by local industry and used as a lignocellulosic matrix to improve the structural integrity and performance of the pellets. Approximately 30 kg of wood powder were used in the preparation of the mixtures.

### Pellets production

Pellets were produced using different proportions of sewage sludge and wood powder: 100% wood, 75% wood/25% sludge, 50% wood/50% sludge, 25% wood/75% sludge, and 100% sludge.

The densification process was performed using a Rotex Master pellet mill, with a nominal production capacity of 1,500 kg·h⁻^1^. During pelletization, the feedstock was subjected to high mechanical pressure and elevated temperature (~ 95 °C), which enhanced interparticle bonding through lignin softening and the physicochemical reorganization of sludge constituents.

Following compaction, the pellets were cooled under controlled conditions to promote mechanical stability, durability, and structural integrity. This stage is critical for the consolidation of interparticle bonds and for improving the overall quality and resistance of the pellets.

After pellet production was completed, the chemical and energy properties of the pellets were investigated.

### Physicochemical and energy characterization

The produced pellets were characterized according to standardized methodologies in order to evaluate their suitability as solid biofuels. The analyses included determination of moisture content, ash content, volatile matter, fixed carbon, higher calorific value (HHV), elemental composition, thermal behavior, and gas emissions.

### Determination of chemical and energy properties

#### Proximate analysis

Moisture content was determined according to ASTM D3173-85 by drying approximately 1 g of sample at 105 °C until constant mass. Ash content was determined following the methodology described by Sánchez et al. (2009), with combustion at 815 °C for 30 min in a muffle furnace. Volatile matter was determined under inert conditions at 900 °C using the same reference method. Fixed carbon content was calculated by difference, according to ABNT NBR 8299 (1983).

#### Elemental analysis (TXRF)

The elemental composition of the samples was determined using Total Reflection X-ray Fluorescence (TXRF), a technique widely applied for multi-elemental analysis at trace levels. The analyses were performed using a S2 PICOFOX™ spectrometer (Bruker), installed at the Central Analytical Laboratory of UNIOESTE (Toledo, Brazil). Samples were homogenized using vortex agitation followed by ultrasonic bath for 20 min to ensure proper dispersion of elements. Aliquots of 10 µL of the sample and internal standard were deposited on quartz carriers and analyzed in triplicate.

It is important to highlight that TXRF provides semi-quantitative to quantitative elemental information, depending on calibration and matrix effects, being highly suitable for comparative analysis of elemental enrichment among samples. Thus, the technique is appropriate for evaluating trends in metal concentration as a function of sludge incorporation.

#### Higher calorific value (HHV)

The higher calorific value was determined using an IKA C2000 bomb calorimeter, following ASTM D2015-66. Combustion was performed under excess oxygen at approximately 30 atm. Calibration was carried out using benzoic acid as a standard.

#### Thermogravimetric analysis (TGA)

Thermogravimetric analyses were conducted using a Perkin Elmer STA 6000 system. The experimental conditions were: Temperature range: 50–600 °C, Heating rate: 20 °C·min⁻^1^, Atmosphere: nitrogen (100 mL·min⁻^1^), Sample mass: 5–10 mg. TGA was used to evaluate thermal stability and decomposition behavior of the pellets.

#### Gas emission analysis (EGA-FTIR)

Gas emissions generated during thermal degradation were analyzed using evolved gas analysis coupled with Fourier transform infrared spectroscopy (EGA-FTIR), employing a PerkinElmer STA 6000 coupled to an FTIR Frontier.

The system was operated under a nitrogen atmosphere with a controlled heating program, enabling the identification of functional groups and the main gaseous compounds released during pyrolysis.

It should be noted that EGA-FTIR provides primarily qualitative and semi-quantitative information on gaseous species. Due to spectral band overlap and the absence of compound-specific calibration, the technique is not intended for accurate quantitative determination. Nevertheless, it is particularly suitable for identifying the presence or absence of potentially toxic compounds and for evaluating emission profiles during thermal degradation.

## Results and discussion

### Pellet production

**Fig. 1:**
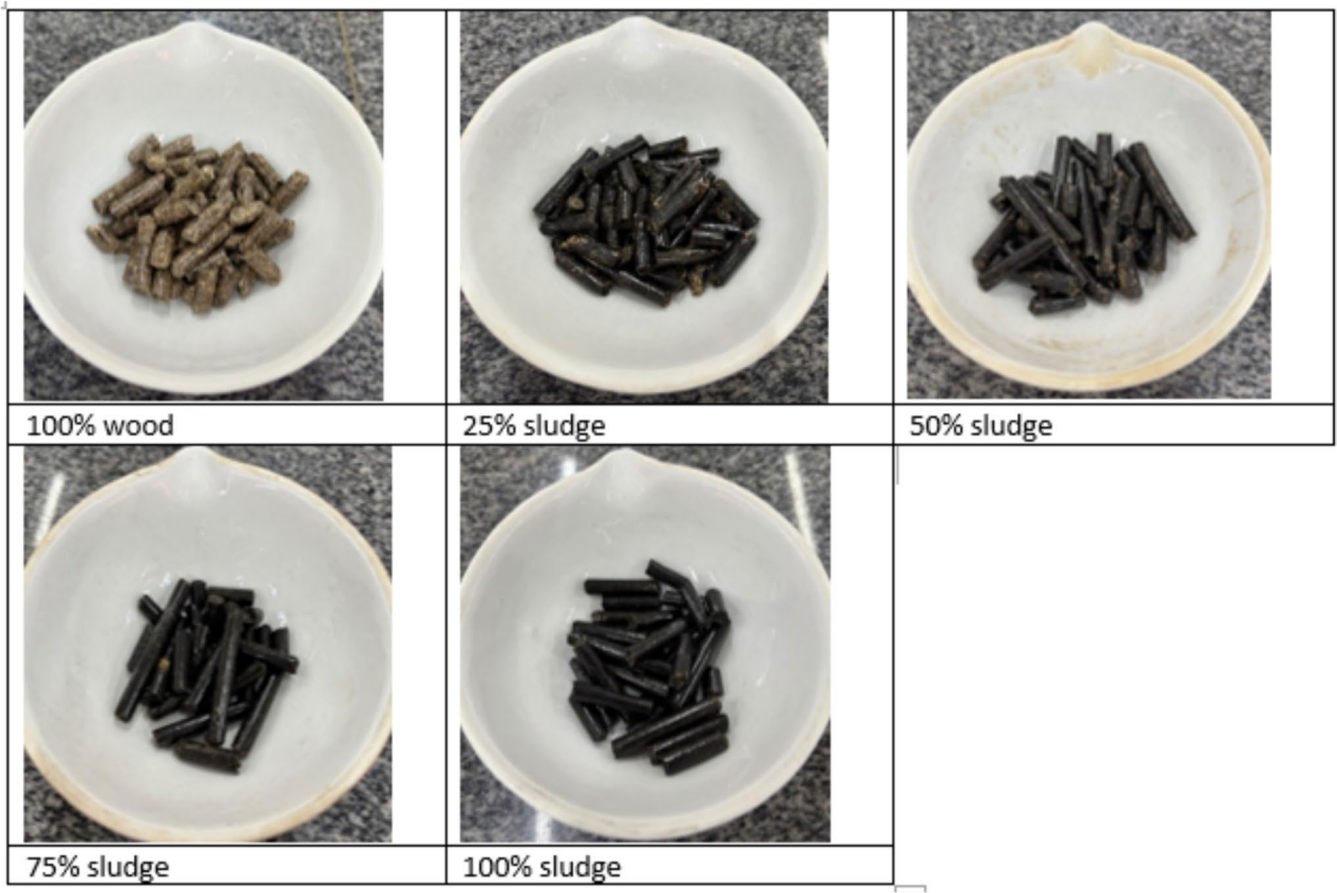
Pellets produced with different proportions of sewage sludge and wood powder: (**a**) PM – 100% wood; (**b**) PL25M – 25% sludge; (**c**) PLM – 50% sludge; (**d**) PL75M – 75% sludge; (**e**) PLE – 100% sludge

Pellets were produced using different proportions of sewage sludge and wood powder, following the procedure described in the methodology (Sect. 2.2), as shown in Fig. 1.

### Proximate analysis

The results of the proximate analysis of the pellets produced with different proportions of sewage sludge and wood powder are presented in Table 1.

**Table 1:** Average values of the immediate analysis of pellets, obtained in triplicate and expressed on dry basis

Material Pellets	Moisture Content (%)	Ash Content (%)	Volatile Material (%)	Carbon Fixed (%)
	ASTM D3173-85	Sánchez et al. (2009)	Sánchez et al. (2009)	NBR 8299
100% Wood	6.76 ± 0.63	0.85 ± 0.41	1.41 ± 0.20	90.97 ± 0.41
25% Sludge 75% wood	3.61 ± 0.90	18.08 ± 061	20.56 ± 3.12	57.74 ± 1.55
50% Sludge 50% wood	2.63 ± 1.13	20.92 ± 3.42	19.02 ± 1.86	57.43 ± 2.14
75% Sludge 25% wood	1.89 ± 0.62	24.43 ± 3.25	21.14 ± 5.09	52.55 ± 2.99
100% sludge	2.17 ± 0.91	29.96 ± 2.78	31.62 ± 1.42	36.25 ± 1.70

The moisture content ranged from 1.89 to 6.76%, remaining within the acceptable limits established by ISO 18134–2 (≤ 10%) for solid biofuels. These values are consistent with those reported in the literature for lignocellulosic biomass and sludge-based fuels, indicating that the drying and preparation procedures adopted in this study were adequate for pellet production.

Ash content showed a significant increase with the incorporation of sewage sludge, ranging from 0.85% for 100% wood pellets to 29.96% for 100% sludge pellets. This behavior is expected due to the high inorganic fraction present in sewage sludge, which includes minerals, salts, and residual metals retained during wastewater treatment processes. Similar trends have been reported in previous studies involving sludge-based fuels, reinforcing the consistency of the results obtained.

Although the ash content exceeds the limits established for commercial wood pellets, it is important to highlight that the objective of this study is not to comply strictly with standards for premium wood pellets, but rather to evaluate the feasibility of waste-derived fuels. In this context, elevated ash content represents a known trade-off associated with the valorization of residues and must be interpreted considering the origin and composition of the material.

The volatile matter content ranged from 1.41 to 31.62%, with lower values compared to typical lignocellulosic biomass reported in the literature. This difference can be attributed to methodological variations, particularly the use of the procedure described by Sánchez et al. (2009), as well as to the higher inorganic content of the sludge, which reduces the fraction of thermally degradable organic compounds.

Fixed carbon content varied between 36.25% and 90.97%, with higher values observed for wood-rich compositions. This parameter is directly related to the combustion behavior and energy release, as fixed carbon contributes to the sustained burning phase. The variation observed reflects the balance between organic and inorganic fractions in the mixtures.

The amount of ash resulting from combustion demonstrates the fraction of the fuel minerals in the oxidized form (Oliveira et al. 2017). According to Obernberger and Thek (2010) the ash content of the raw material does not intervene in the pelletizing process, however, depending on the ash chemical composition, mineral fouling can form damaging the combustion equipment. In Magdziarz and Werle’s studies (2014) a percentage of 32–36% was found in three types of sewage sludge from Poland, which is close to the values of this study, but the amount of ash is related to the composition of the material that varies with origin and region.

Volatile matter comprises compounds that are released as gases when the material is exposed to elevated temperatures (Oliveira et al. 2017). Wattan et al. (2025) reported volatile matter contents ranging from 38.9 ± 0.6% to 82.5 ± 0.6% for pellets produced from torrefied linseed straw at different temperatures. Similarly, Bilbao et al. (2025) reported values of 61.78% for rice husk, 83.08% for wheat straw, 91.16% for corn straw, and 72.1% for sewage sludge. Lower values were observed by Arantes et al. (2008), who found 31% for corn cob and 25% for sugarcane bagasse.

In the present study, volatile matter ranged from 1.41 ± 0.20% to 31.62 ± 0.42% (Table 1). Differences between the values obtained in this study and those reported in the literature may be attributed to methodological variations. Some authors employed standards such as ABNT NBR 8112/86 or alternative procedures for volatile matter determination, with differences mainly related to residence time in the muffle furnace. In contrast, the method adopted in this study followed the procedure described by Sánchez et al. (2009), as detailed in the methodology section.

Fixed carbon represents the non-volatile fraction of carbon remaining after devolatilization and plays a key role in the energy behavior and combustion processes, as it is responsible for a significant portion of heat generation during combustion (Oliveira et al. 2017). The combustion process generally occurs in three overlapping stages: moisture evaporation, pyrolysis with the release and combustion of volatile compounds, and the oxidation of fixed carbon, which predominates in the final stage (Tomeleri et al. 2017).

Wattan et al. (2025) reported fixed carbon values ranging from 13.9 ± 0.4% to 53.0 ± 1.3% for linseed straw pellets. In the study by Bilbao et al. (2025), fixed carbon contents were 8.04% for rice husk, 10.29% for wheat straw, 21.1% for forest residues, and 11.9% for sewage sludge. Pokorná et al. (2009) reported values of 19.7% for thermally treated sludge, 8.3% for dehydrated digested sludge, and 18.4% for dried activated sludge; however, these values were obtained using thermogravimetric analysis (TGA).

In the present study, fixed carbon values ranged from 36.25 ± 1.70% to 90.97 ± 0.41% (Table 1). The determination of fixed carbon was carried out according to the ABNT NBR 8299 standard, which may explain differences in comparison with other studies, particularly those employing techniques that do not explicitly account for moisture content. In addition to methodological differences, variations in fixed carbon content can also be attributed to the intrinsic characteristics of the raw materials, such as their chemical composition, degree of organic matter stabilization, ash content, and prior thermal or biological treatment, all of which directly influence the carbonization behavior and residual carbon fraction.

### Elemental composition (TXRF analysis)

The elemental analysis revealed a progressive increase in the concentration of most elements as the proportion of sewage sludge increased in the pellets. This trend is consistent with the known composition of sewage sludge, which is typically enriched in metals, phosphorus, sulfur, and mineral components. The concentrations of the main elements are presented in Table 2.

**Table 2:** Comparison of elementary contents (mg.kg⁻^1^) obtained by TXRF and literature values for wood pellets and dry sewage sludge

Element	0%Sludge (Wood)	50% Sludge	100% Sludge	Range Wood	Sewage Sludge band
Sodium	255	427	928	50–300	800–4000
Chromium	3.4	143	153	1–10	50–500
Manganese	134	288	378	50–200	200–800
Iron	1599	35,293	51,789	100–1000	20,000–80000
Cobalt	0.4	92	131	< 1	20–200
Nickel	2.3	24	29	1–10	20–200
Copper	6.2	222	276	2–20	200–600
Zinc	20.6	1940	2117	10–100	1000–5000
Arsenic	0.5	12.7	13.0	< 1	10–30
Cadmium	20	34.8	153	< 1	1–50
Lead	0.25	13.9	22.7	< 1	10–100
Calcium	1852	14,113	16,321	500–2000	10,000–25000
Phosphorus	30	533	535	10–100	300–1000
Sulphur	49	5440	6543	100–500	5000–15000
Potassium	595	757	1050	500–2000	500–3000
Chlorine	7	81	88	10–100	100–1000
Aluminum	180	1866	1923	100–500	1000–4000
Titanium	13.6	227	410	1–30	200–600

A clear increase in the concentration of most elements was observed with increasing sewage sludge content in the pellets. This trend is consistent with the intrinsic characteristics of sewage sludge, which is typically enriched in metals, phosphorus, sulfur, and mineral salts retained during wastewater treatment processes (Fytili and Zabaniotou 2008; Gusiatin and Rouhani 2023).

The concentration ranges adopted for comparison were based on literature data reported by Fytili and Zabaniotou (2008), Gusiatin and Rouhani (2023), Mtshali et al. (2014), and Thiengo et al. (2023). Most of the elements analyzed in this study fall within the expected ranges for both biomass and sewage sludge. However, cadmium concentrations exceeded the typical values reported in the literature, which may be associated with differences in sludge origin and anthropogenic contributions, such as industrial effluents and urban contamination. As reported by Fuentes et al. (2004), the metal content in sewage sludge is strongly influenced by the source of the wastewater and the treatment processes applied.

**Fig. 2:**
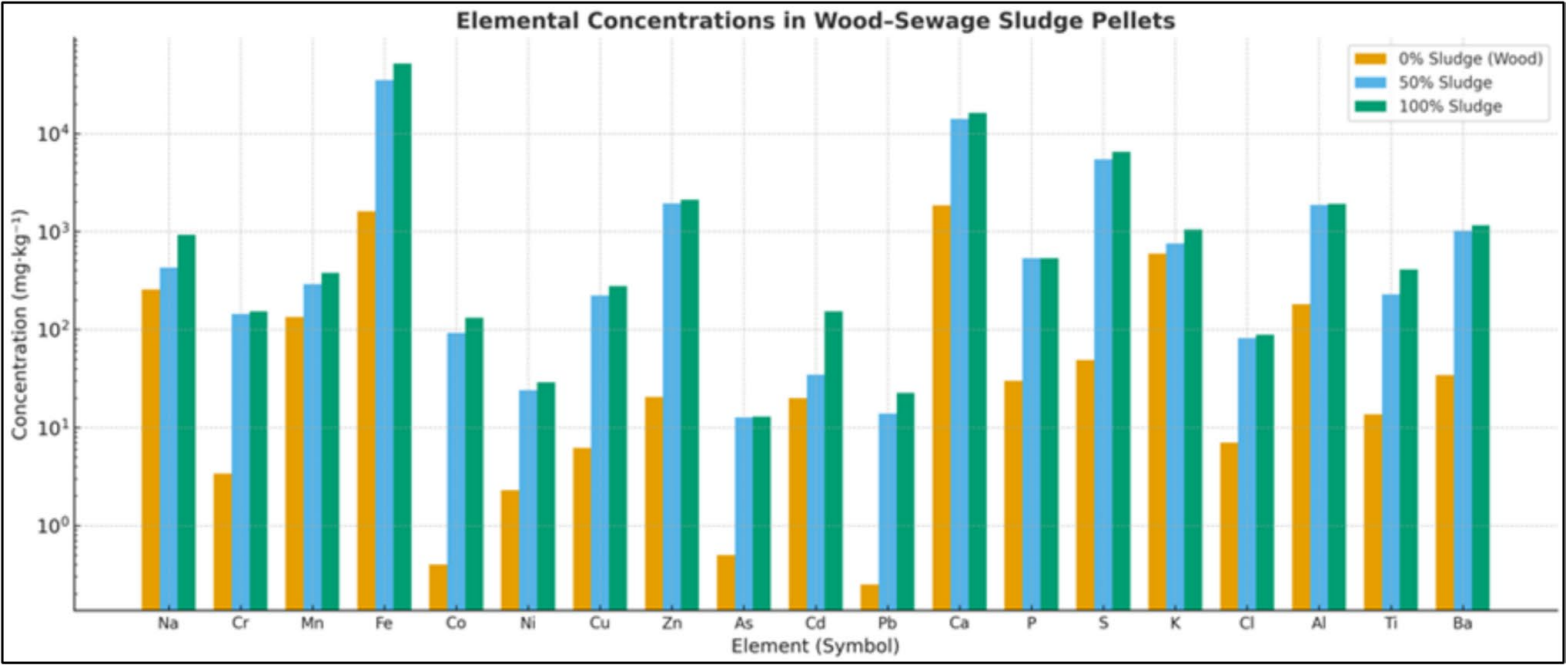
Comparative chart of elemental concentration by TXRF in composite pellets (0%, 50% and 100% sewage sludge)

Figure 2. Comparison of elemental concentrations (mg·kg⁻^1^) obtained by TXRF for pellets with varying proportions of sewage sludge (0%, 50%, and 100%). Data are displayed on a logarithmic scale. The results show a systematic increase in inorganic elements, particularly Fe, Al, Ca, Zn, Cu, and S, as the sludge content increases, reflecting the contribution of mineral and metallic constituents from sewage sludge.

From an environmental perspective, the presence of heavy metals requires careful consideration, particularly regarding emission control and ash management during combustion. However, these limitations do not preclude the use of sludge-derived fuels, provided that appropriate technologies and regulations are applied.

### Higher calorific value (HHV)

The higher calorific value (HHV) of the pellets ranged from 14.45 to 19.01 MJ·kg⁻^1^. As expected, HHV decreased with increasing sludge content, reflecting the lower organic carbon content and higher mineral fraction of the sludge.

Pellets containing up to 50% sludge exhibited HHV values above 16.56 MJ·kg⁻^1^, meeting the requirements established by ISO 18125 for commercial wood pellets. This result is particularly relevant, as it demonstrates that partial substitution of wood by sewage sludge does not compromise the energy performance required for practical applications.

The obtained values are comparable to those reported for various types of biomass and waste-derived fuels, confirming the potential of sewage sludge as a viable component in solid biofuels. These findings reinforce the concept of co-processing residues with lignocellulosic biomass to optimize energy properties. The results are presented in Table 3.

**Table 3:** Upper calorific value, measured in Adiabatic Calorimetric Pump, ASTM D2015-66 standard

Material Pellets	Calorific value (MJ kg ^−1^)	Calorific Value (kWh.kg ^−1^)
100% Wood	19.006 ± 0.21	5.279
75% Wood 25% Sludge	16.938 ± 0.19	4.705
50% Wood 50% Sludge	16.543 ± 0.11	4.595
25% Wood 75% Sludge	15.866 ± 0.23	4.407
100% Sludge	14.447 ± 0.21	4.013

Pellets produced from a wide range of raw materials have been reported in the literature, and the composition and nature of these materials play a key role in determining their calorific value. Pradhan et al. (2025) reported distinct calorific values for pellets derived from three types of Canadian biomass—hardwood, coniferous wood, and wheat straw—of 18.00 MJ·kg⁻^1^, 18.07 MJ·kg⁻^1^, and 16.07 MJ·kg⁻^1^, respectively.

According to Wzorek (2012), solid fuels should exhibit a higher heating value (HHV) above 13 MJ·kg⁻^1^ to be considered suitable for energy applications. In addition, ISO 18125:2017 recommends a minimum value of 16.56 MJ·kg⁻^1^ for the commercial classification of pine wood pellets.

Sermyagina et al. (2022) demonstrated that hot water treatment for the extraction of hemicelluloses from wood can enhance the energy content of sawdust pellets by approximately 3%, reaching values of up to 20.62 MJ·kg⁻^1^. Similarly, Wattan et al. (2025) reported that pellets produced from torrefied linseed straw exhibited a significant increase in calorific value compared to untreated material. While the untreated biomass presented a calorific value of 19.7 MJ·kg⁻^1^, torrefaction at temperatures between 250 and 300 °C increased this value to a range of 26.6–28.6 MJ·kg⁻^1^. This improvement was primarily attributed to the increased carbon content of the thermally treated material.

Wzorek et al. (2021), presented the possibility of valorization and use of animal manure (camel and cow) (9.2–10.4 MJ kg ^−1^) mixing it with agro-industrial biomass (cotton stalk and canola cake) (19.5–24.5 MJ kg ^−1^) for the purpose of producing pellets for use in power generation processes. In his studies the results showed that the addition of agro-industrial biomass (even in 10%) to animal manure changed the specific combustion parameters. From the mixtures of these biomasses these authors obtained pellets with calorific value ranging from 17 to 20 MJ kg ^−1^.

The acceptable calorific value of animal waste is approximately 7–8 MJ kg ^−1^, to be considered an interesting co-fuel for waste-to-energy conversion boilers (Moravian et al. 2013).

Apiculture residue pellets (slumgum) have a calorific value of 27.66 MJ kg ^−1^ due to its high fat content (40.25%) (Bilbao et al. 2025). The research by Güler (2024) highlights the potential of tea waste pellets also as a sustainable biofuel, with 18.5 MJ kg ^−1^.

For pellets made of pine sawdust mixed with wheat and canola straw, calorific value was higher than that recommended by ISO 17225–6:2014 for woody biomass (≥ 16.5 MJ kg^−1^) and non-woody biomass (≥ 14.5 MJ kg^−1^) (Stasiak et al. 2017).

The results obtained in this project for calorific value were between 14.447 and 19.006 MJ kg ^−1^. Thus, all the pellets, are within the limits of values recommended by ISO 17225–6:2014, woody biomass, non-woody and its mixtures, in addition to meeting also the recommended by Wzorek (2012) so that a solid material can be used as fuel must have a calorific value above 13 MJ kg ^−1^. In order to meet ISO 18125:2017, which suggests a value greater than or equal to 16.56 MJ kg ^−1^, it is necessary to add at least 50% of wood to the sewage sludge.

### Thermogravimetric analysis (TGA)

Thermogravimetric analysis revealed distinct thermal degradation behaviors depending on pellet composition. Three main stages of mass loss were identified: moisture evaporation (up to ~ 120 °C), devolatilization (200–380 °C), and residual degradation (above 400 °C).

Wood-rich pellets exhibited higher mass loss during the devolatilization stage, associated with the decomposition of hemicellulose and cellulose. In contrast, sludge-rich pellets showed slower degradation rates and higher residual mass, indicating increased thermal stability due to the presence of inorganic components.

**Fig. 3:**
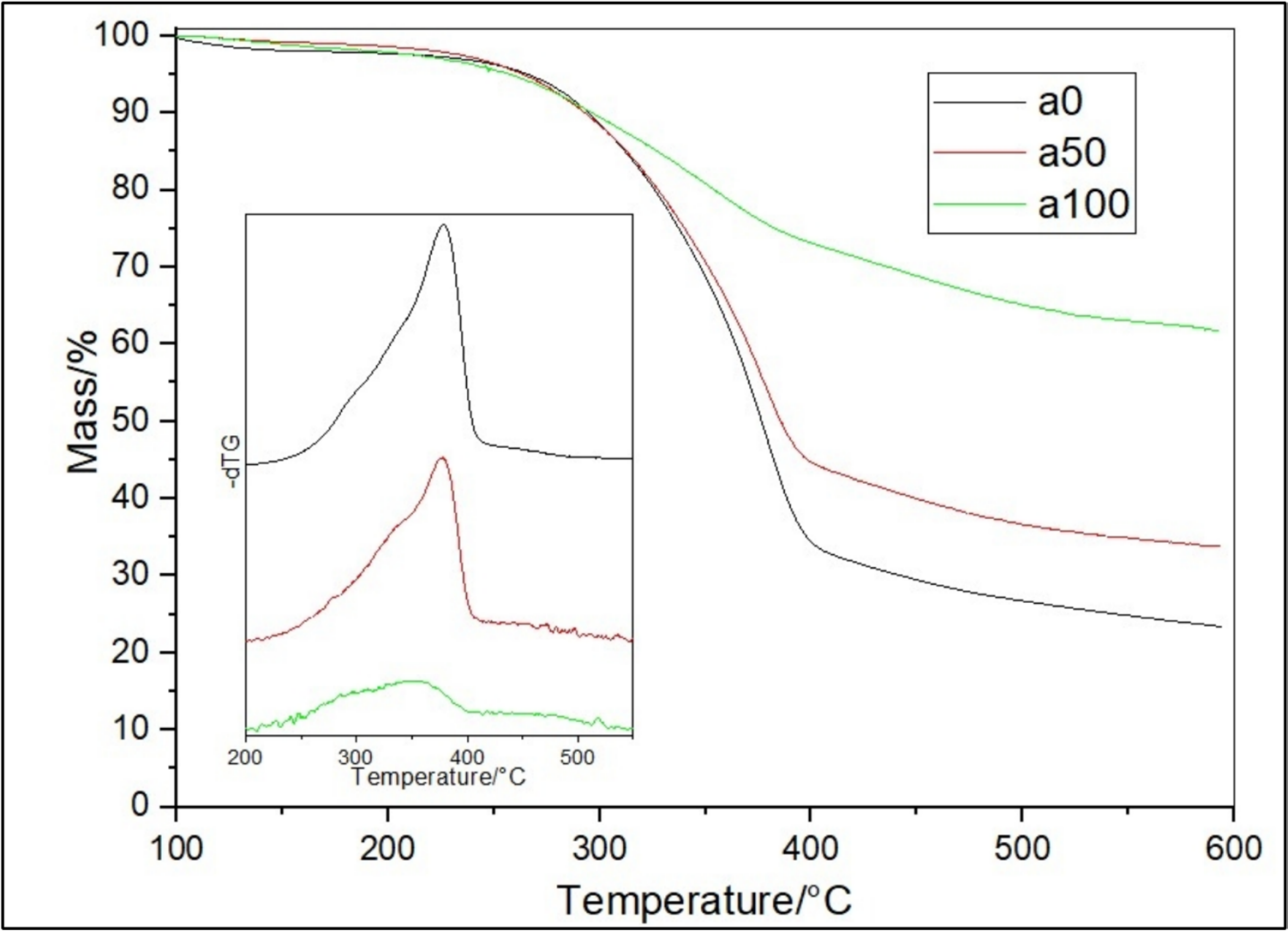
TGA curves of PLE (A100), PLM (A50) and PM (A0) in nitrogen

Thermogravimetric analysis (TGA) of the pellets enabled the evaluation of their thermal decomposition behavior, as well as the identification of temperature ranges in which mass loss was more pronounced. Figure 3 shows the TGA curves of pellets composed of (A100) 100% sewage sludge (PLE), (A50) 50% sludge and 50% wood (PLM), and (A0) 100% wood (PM), over a temperature range from 25 to 900 °C under a nitrogen (N₂) atmosphere.

At temperatures between 25 and 120 °C, a slight mass loss was observed in all samples, which is attributed to the evaporation of surface moisture (approximately 2–6%). The PM sample (A0) exhibited a higher mass loss in this initial stage, possibly due to its greater moisture content, corroborating the values obtained according to the ASTM D3173-85 standard used in this study. In this temperature range, PLM (A50) and PLE (A100) showed similar mass loss behavior up to approximately 300 °C.

Between 200 and 380 °C, the most significant mass loss occurred, as evidenced by the steepest slope in the thermogravimetric curves. This stage is primarily associated with the decomposition of hemicellulose (200–320 °C) and cellulose (320–380 °C), in agreement with Apaydin, Varol and Mutlu (2023). The more pronounced mass loss observed for PM (A0) in this region reflects its higher content of volatile, easily degradable lignocellulosic compounds, which enhances its reactivity under thermal conditions. In contrast, PLE (A100) exhibited a broader and less intense degradation profile, indicating slower decomposition kinetics and greater thermal stability, mainly due to its higher inorganic fraction.

At temperatures above 400 °C, the degradation rate decreased, indicating the progressive decomposition of more thermally stable compounds, particularly lignin, along with the formation of char and mineral residues. The persistence of lignin-derived structures, associated with aromatic and phenolic compounds, is consistent with the observations reported by Yang et al. (2023). The higher residual mass observed for PLE (A100) confirms its elevated ash and mineral content, whereas PM (A0), composed predominantly of organic matter, resulted in significantly lower residue formation.

From a thermochemical perspective, the observed behavior reflects the intrinsic composition of the materials: lignocellulosic biomass (PM) favors devolatilization and energy release, while sewage sludge (PLE), due to its inorganic constituents, exhibits enhanced thermal resistance and greater solid residue formation. Accordingly, the thermal stability followed the order PLE (A100) > PLM (A50) > PM (A0), whereas the ease of degradation and volatile content followed the inverse trend: PM (A0) > PLM (A50) > PLE (A100).

These findings are consistent with Nath et al. (2024), who reported that higher volatile fractions result in mass losses exceeding 70% during pyrolysis. Similarly, Apaydin, Varol and Mutlu (2023) demonstrated that lignocellulosic materials rich in hemicellulose and cellulose exhibit higher mass losses, whereas materials with higher ash and lignin contents tend to produce more thermally stable residues. In addition, Yang et al. (2023) observed similar behavior in coal dust, where variations in volatile and inorganic fractions significantly influenced mass loss patterns.

In the present study, PM (A0) showed the highest total mass loss, reaching approximately 75%, while PLE (A100) exhibited the lowest mass loss (approximately 39%) under an inert nitrogen (N₂) atmosphere. This difference has direct implications for energy applications, as higher mass loss is generally associated with greater release of volatiles and higher energy conversion efficiency during thermochemical processes such as pyrolysis and combustion.

Nath et al. (2024) also investigated the thermal behavior of pellets with different compositions, including 100% wheat straw and a blended formulation containing additives. They reported three main degradation stages: drying, devolatilization (the most significant stage, with approximately 65% mass loss between 150 and 550 °C), and carbonization. In comparison, the present study showed a more concentrated degradation range between 200 and 450 °C, indicating differences in composition and thermal reactivity.

According to Magdziarz and Werle (2014), moisture evaporation typically occurs up to 150 °C, while the pyrolysis of sewage sludge begins above 200 °C and extends to approximately 540 °C. These observations are consistent with the results obtained in this study and reinforce the reliability of the thermal behavior identified.

Finally, from an application standpoint, the incorporation of sewage sludge into biomass pellets (PLM) represents a compromise between thermal stability and energy efficiency, suggesting that co-processing strategies may optimize both fuel performance and waste valorization.

### Gas emission analysis (EGA-FTIR)

**Fig. 4:**
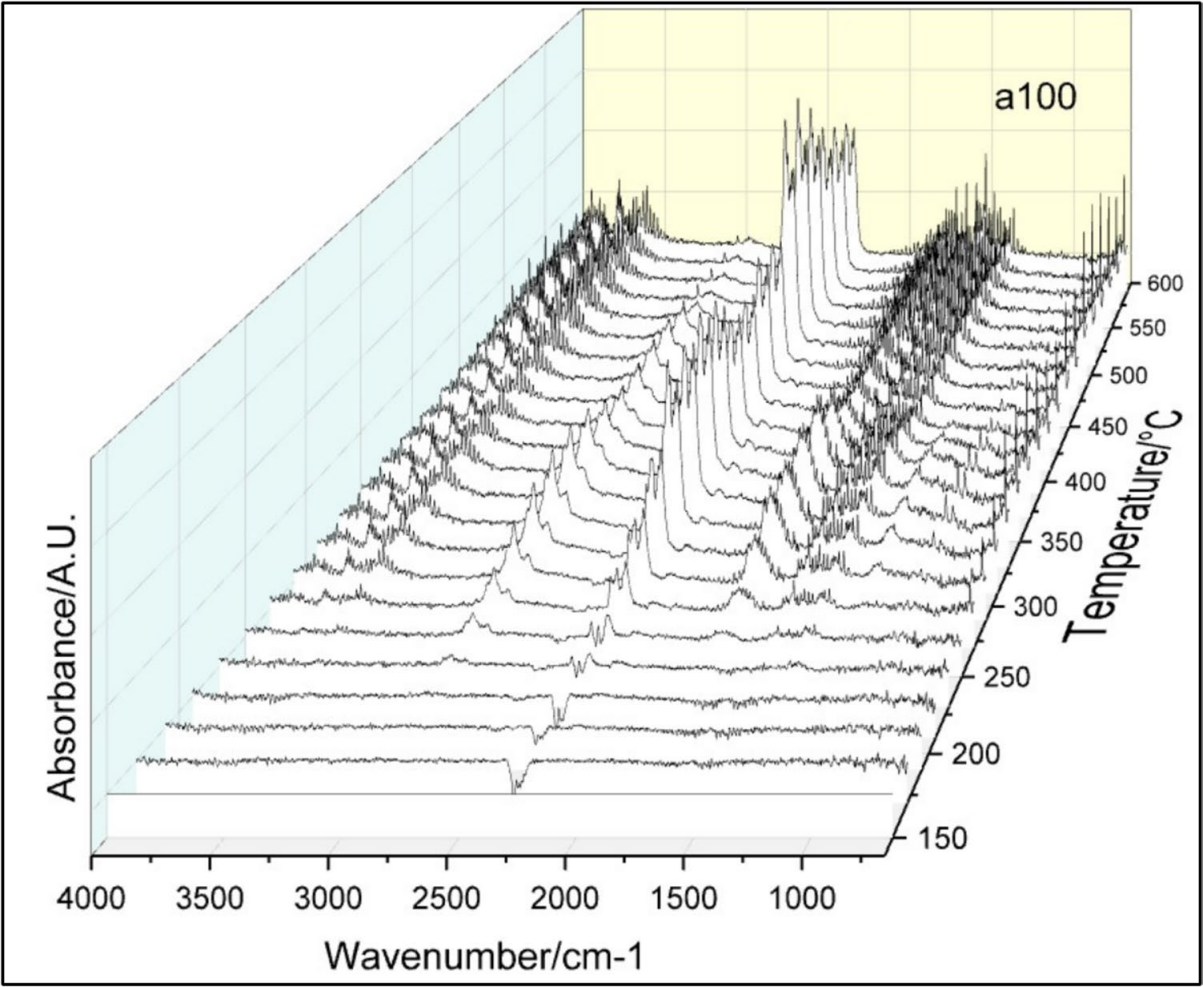
Three-dimensional EGA-FTIR spectrum of PLM thermal degradation gases (A50) at a heating rate of 20 °C min ^−1^;N_2_ flow of 20 mL min^−1^

**Fig. 5:**
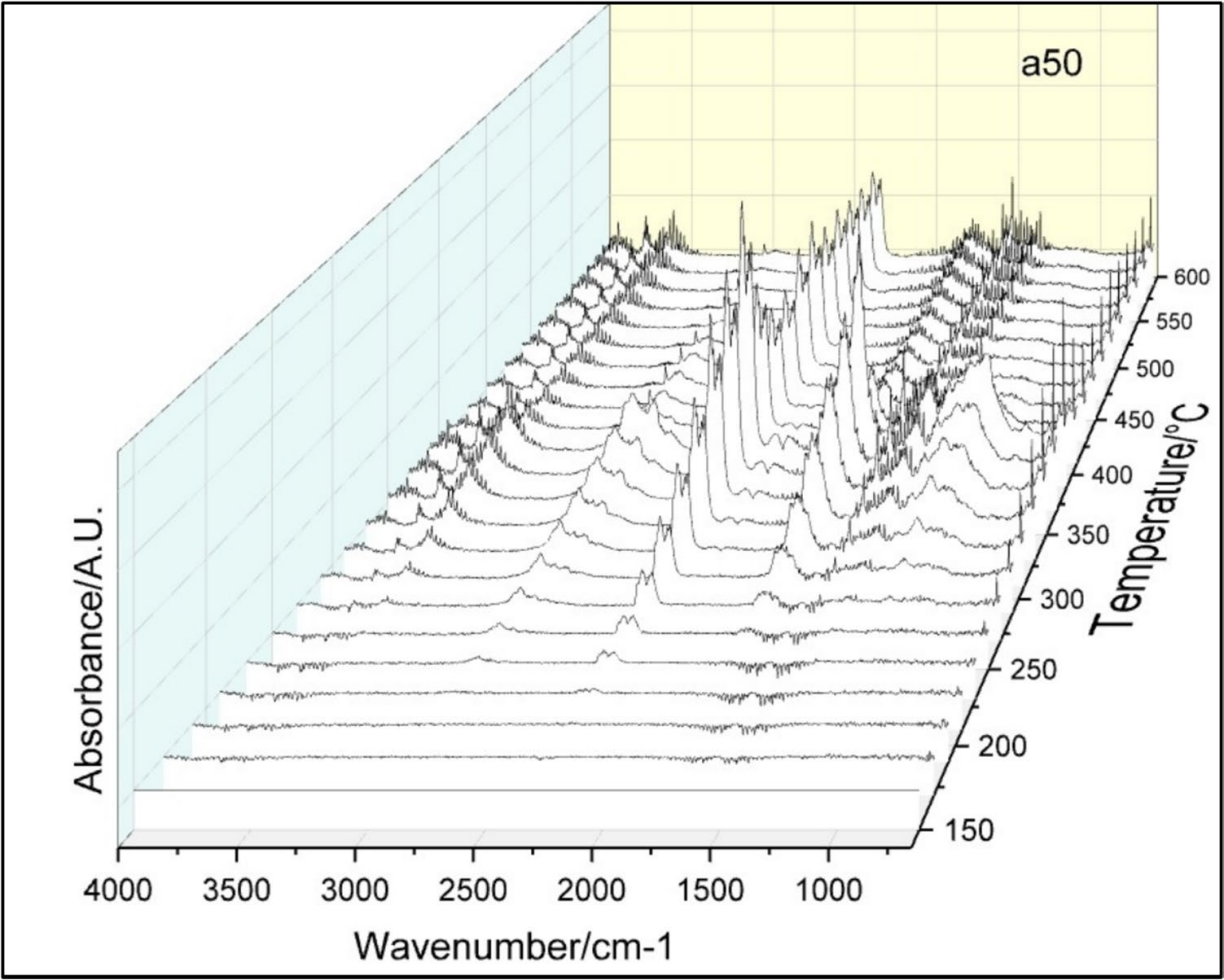
Three-dimensional EGA (TG-FTIR) spectrum of PM pyrolysis (A0) at a heating rate of 20 °C min^−1^; N2 flow rate of 20 mL min^−1^

**Fig. 6:**
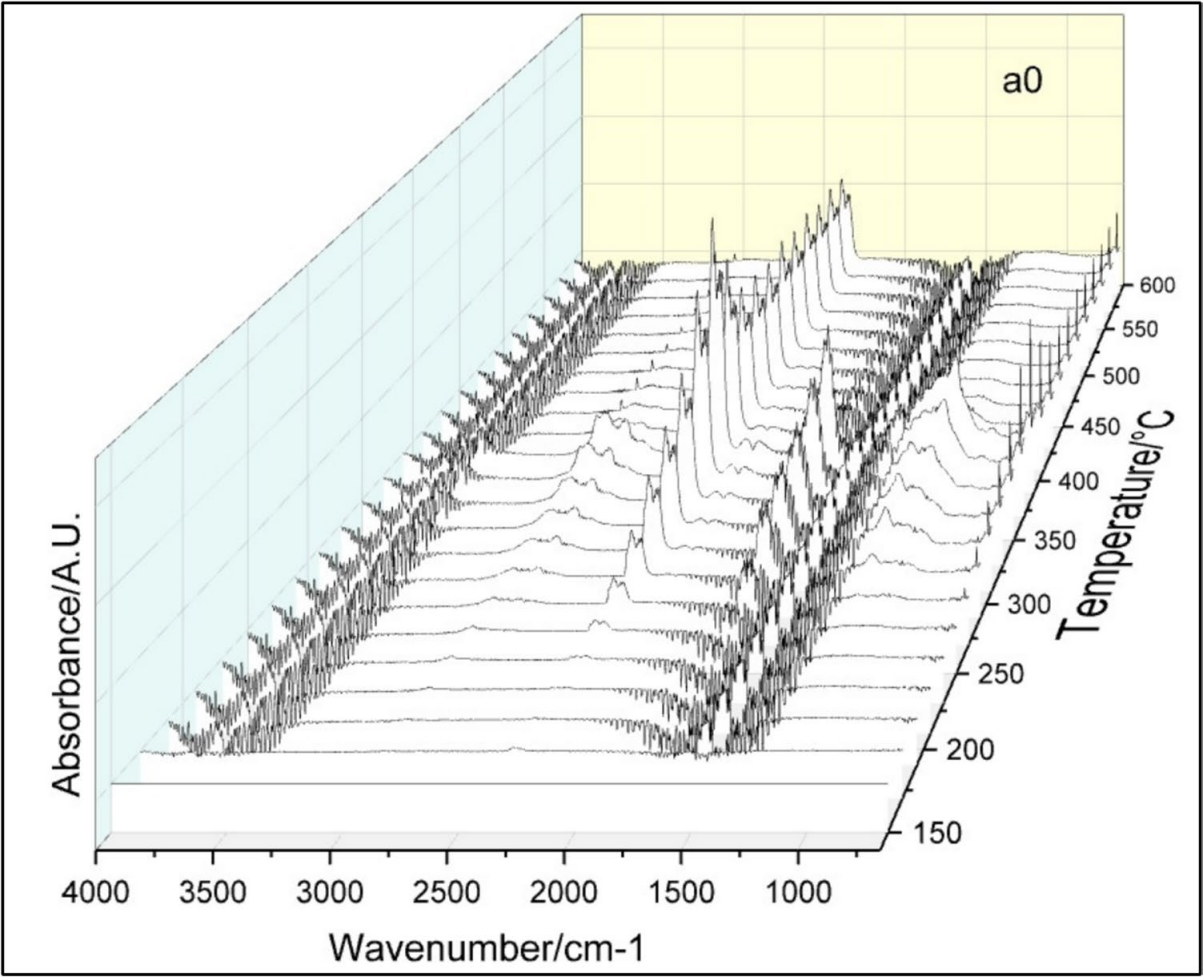
Three-dimensional EGA (TG-FTIR) spectrum of PM pyrolysis (A0) at a heating rate of 20 °C min−1; N2 flowrate of 20 mL min^−1^

Thermogravimetric analysis (TGA) combined with evolved gas analysis (EGA-FTIR) provided a comprehensive understanding of the thermal decomposition behavior and gaseous emissions of the pellets. Figure 2 presents the mass loss profiles, while Figs. 4, 5, and 6 show the three-dimensional FTIR spectra of the evolved gases, and Fig. 6 summarizes the main functional groups identified in the gaseous phase.

**Fig. 7:**
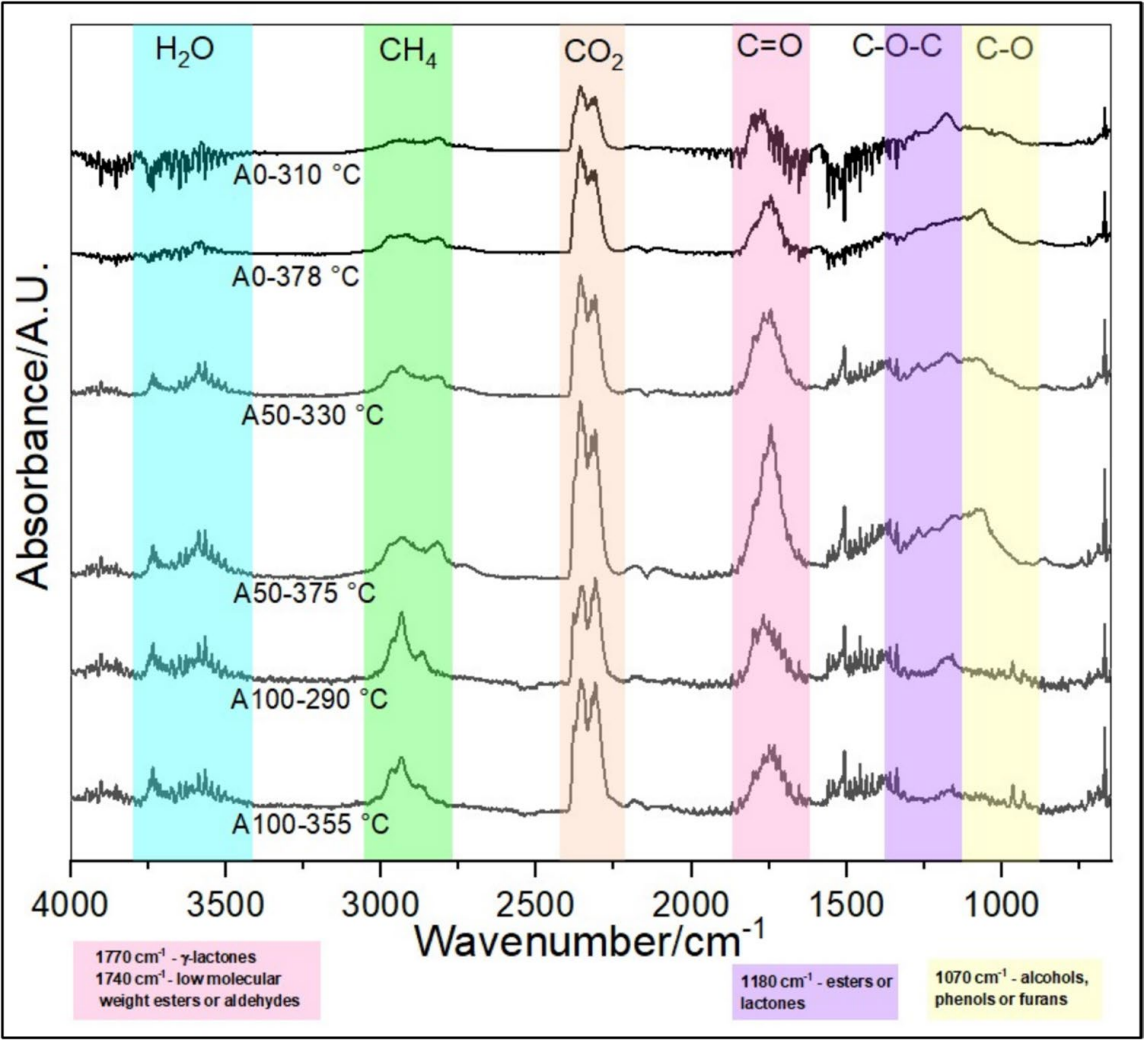
EGA chart (FTIR coupled evolved gases analysis) Absorption spectrum in the infrared region for pellets

In the initial stage (25–120 °C), a slight mass loss was observed for all samples (Fig. 3), which corresponds to the evaporation of surface moisture. This process is confirmed by the EGA results (Fig. 7), where a broad absorption band between 3700 and 3200 cm⁻^1^ indicates the release of water vapor (H₂O), in agreement with Hernandez, Okonta and Freeman (2017).

The main devolatilization stage occurred between 200 and 380 °C (Fig. 3), where the highest mass loss rates were observed, particularly for PM (A0). This behavior is directly correlated with intense gas evolution in the same temperature range (Figs. 4, 5, 6), mainly associated with CO₂ (≈2350 cm⁻^1^) and CO formation, resulting from the degradation of hemicellulose and cellulose, as also reported by Apaydin, Varol and Mutlu (2023). The PM (A0) sample exhibited the most pronounced gas release, reflecting its higher content of volatile organic compounds and lignocellulosic structures.

In contrast, PLE (A100) showed a broader and less intense gas evolution profile (Fig. 4), indicating slower degradation kinetics and greater thermal stability, consistent with its higher inorganic and ash content. The PLM (A50) sample presented intermediate behavior (Fig. 5), with gas evolution peaks occurring at slightly higher temperatures (~ 330–375 °C), suggesting a synergistic interaction between wood and sewage sludge that promotes more controlled thermal decomposition.

The correspondence between TGA and EGA is further evidenced by the alignment of peak degradation temperatures. For PLE (A100), the main gas evolution events occurred at approximately 290 °C and 355 °C (Fig. 4), while for PLM (A50) they were observed at approximately 330 °C and 375 °C (Fig. 5), and for PM (A0) at approximately 310 °C and 378 °C (Fig. 6). These temperatures are consistent with the maximum degradation rates observed in the DTG curves (Fig. 3), confirming the reliability of the thermal analysis.

At temperatures above 400 °C, the TGA curves (Fig. 3) show a reduction in mass loss rate, indicating the transition to the carbonization stage. This stage is accompanied by the continued release of gases such as CH₄ (≈3016 cm⁻^1^) and CO (Fig. 7), associated with lignin decomposition and secondary reactions involving char formation. The persistence of gas evolution at higher temperatures reflects the greater thermal resistance of lignin and the complexity of secondary pyrolytic reactions, as discussed by Yang et al. (2023).

The FTIR spectra (Fig. 7) also revealed the presence of carbonyl (C = O, 1770–1740 cm⁻^1^) and C–O/C–O–C groups (1180–1070 cm⁻^1^), indicating the formation of oxygenated compounds such as aldehydes, phenols, ethers, and furans. These compounds are typical products of lignocellulosic and sludge pyrolysis and are consistent with the findings of Miskolczi and Tomasek (2022), Hu et al. (2019), and Wang et al. (2020).

From a thermochemical standpoint, the results demonstrate that PM (A0) exhibits higher reactivity and greater release of volatile gases, resulting in higher mass loss (~ 75%), whereas PLE (A100) shows lower mass loss (~ 39%) and higher residue formation, due to its inorganic composition. The PLM (A50) sample presents intermediate behavior, combining relatively high volatile release with improved thermal stability, which may be advantageous for controlled energy conversion processes.

From an environmental perspective, the identification of gases such as CO, CH₄, and oxygenated organic compounds highlights the importance of emission control strategies during thermochemical conversion. As reported by Hernandez, Okonta and Freeman (2017) and Hu et al. (2019), many of these compounds present toxicity and may pose risks to human health. Therefore, technologies such as co-pyrolysis, catalytic upgrading, and gas treatment systems are essential to minimize environmental impacts.

Overall, the integration of TGA and EGA analyses provides a robust understanding of the relationship between material composition, thermal behavior, and gaseous emissions, reinforcing the potential of co-processed pellets (PLM) as a balanced alternative between energy efficiency and environmental performance.

However, it should be emphasized that the assignment of specific toxic compounds based solely on EGA-FTIR analysis is limited, and a more accurate identification would require complementary.

## Conclusion

This study conclusively demonstrated the technical feasibility of producing pellets from sewage sludge and Pinus wood powder mixtures, supporting their application as a sustainable solid biofuel within a waste valorization approach. The results confirmed that sewage sludge can partially replace lignocellulosic biomass without compromising the energetic applicability of the pellets, particularly in formulations containing up to 50% sludge. Among the evaluated compositions, the 50:50 sludge-to-wood ratio presented the most suitable balance between calorific value, thermal stability, and ash content, while remaining compatible with conventional wood pellet combustion conditions.

The physicochemical, thermogravimetric, and EGA-FTIR analyses demonstrated that sludge incorporation increases mineral content and thermal stability, without generating critical levels of toxic gaseous emissions under the evaluated conditions. Although the increase in heavy metal concentrations highlights the need for proper environmental management and emission control, these factors do not prevent the energetic utilization of sewage sludge when appropriate monitoring strategies are adopted.

Overall, the findings reinforce the potential of sewage sludge as a viable alternative to landfill disposal, contributing simultaneously to energy recovery, reduction of environmental impacts, lower dependence on virgin raw materials, and the promotion of circular economy principles. Therefore, the conversion of sewage sludge into pelletized biofuel represents a technically and environmentally promising strategy for sustainable waste management and renewable energy generation.

## Data Availability

The authors declare that the data supporting the findings of this study are available within the paper and its Supplementary Information files. Should any raw data files be needed in another format they are available from the corresponding author upon reasonable request. Source data are provided with this paper.
